# Homeless people: a review of personality disorders

**DOI:** 10.3389/fpsyt.2024.1362723

**Published:** 2024-05-08

**Authors:** Joana Henriques-Calado, João Gama Marques

**Affiliations:** ^1^ Centro de Investigação em Ciência Psicológica (CICPSI), Faculdade de Psicologia, Universidade de Lisboa, Lisboa, Portugal; ^2^ Faculdade de Psicologia, Universidade de Lisboa, Alameda da Universidade, Lisboa, Portugal; ^3^ Consulta de Esquizofrenia Resistente, Hospital Júlio de Matos (HJM), Unidade Local de Saúde São José (ULSSJ), Centro Clínico Académico de Lisboa (CCAL), Lisboa, Portugal; ^4^ Clínica Universitária de Psiquiatra e Psicologia Médica (CUPPM), Faculdade de Medicina, Universidade de Lisboa (FMUL), Centro Académico de Medicina de Lisboa (CAML), Lisboa, Portugal; ^5^ Homeless Outreach Psychiatric Engagement for Lisboa (HOPE 4 Lisboa), Santé Mentale et Exclusion Sociale (SMES) Europa, Lisboa, Portugal

**Keywords:** homeless, personality, psychiatry, psychology, psychopathology, mental health

## Abstract

Personality disorders in homeless people pose a challenge to the medical community and society, requiring specialized approaches for these super-difficult patients. The prevalence of personality disorders is higher in homeless populations than in the general population. However, there is a knowledge gap regarding personality disorders among people experiencing homelessness, and the implications of this lack of recognition are substantial. This paper provides a brief narrative review of personality disorders among homeless individuals. The primary importance and specificity of these disorders in this population remain unexplored. We searched PubMed and Web of Science databases in February and November 2023 using the keywords ‘homeless’ and ‘personality disorder’, and selected fifty-eight studies to be included in this literature review. The main themes of the results were personality disorders in homeless individuals and comorbid psychiatric disorders; risk factors and other psychological and behavioral data; clinical and intervention outcomes; and challenges linked to assessment, treatment, and intervention. The homeless population experiences significant diagnostic variability and the diagnosis of personality disorders is still evolving, contributing to difficulties in diagnosis, assessment, and treatment. A future challenge is to raise clinical awareness and optimize research knowledge, assessment, and intervention in personality disorders among homeless individuals with comorbid psychiatric disorders.

## 1 Introduction

Personality disorders (PD) among the homeless pose a challenge to medicine and society and are many times framed as difficult or super-difficult patients. Difficult, because they are prevalent in primary care settings, have more psychiatric disorders, functional impairment, health care utilization, and dissatisfaction with care (1). Super difficult, because besides all that, they are homeless, living and dying on the streets, neglected by society, lacking the appropriate health care from community psychiatry (2).

PD’s affect more than 10% of the population but are widely ignored by health professionals due to the associated stigmas (3). However, available data remain scattered; two recent meta-analyses reported varying prevalence estimates for lifetime PD of 25.4% (4) and, around 7.8% (5). Studies have shown that PD causes considerable morbidity, is associated with high service and societal costs, and usually has an adverse effect on the progress in the treatment of other psychiatric disorders (6). Challenges also include difficulty in approaching the patient because of poor pharmacological results and a significant treatment abandonment rate (7). According to some experts, PD should be recognized as a psychiatric priority and a major condition in mainstream psychiatry across the world (5, 6). The principal challenge of the 21st century is determining the most efficient treatment for PD (7).

The prevalence of PD’s is much higher in homeless individuals than in the general population (7–9). A recent systematic review (10) highlighted that PD is very common in homeless individuals, with frequencies ranging from 64% to 79% for any PD. Some authors (9, 11, 12) have drawn attention specifically to the gap in knowledge about PD in individuals experiencing homelessness based on the absence of reliable and valid PD diagnoses. The implications of this lack of recognition of PD and the limited data about them in homeless populations are substantial (8, 9).

Research on PD in homeless people is limited. This article briefly reviews the existing literature on PD in homeless population and intends to address the existing data based on the state-of-the-art research topic.

## 2 Methods

Our research was conducted with the terms ‘homeless’ and ‘personality disorder’, in searches managed on PubMed (search details: (homeless*[Title/Abstract]) AND (personality disorder [Title/Abstract])), and on Web of Science (search details: (homeless*[Title]) AND (personality disorder [Title])) – both without any time limit. On February 20^th^ and November 17^th^, 2023, the results yielded 65 articles on PubMed and 66 articles on Web of Science based on the above-mentioned keywords. A book was included following a manual search. The two authors served as evaluators. Of the 131 entries, considering the exclusion criteria of duplicates and articles unrelated to the topic. Only English and French documents (with abstracts in English) were considered. Finally, a total of 58 articles were subjected to analysis in this narrative review.

## 3 Results

### 3.1 Personality disorders in patients living homeless and comorbid psychiatric disorders

Fazel’s (8) systematic review and meta-regression analysis, drawing on data from 5684 homeless individuals, reported the prevalence of PD among the homeless in Western countries as 23.1% (CI 15.5%–30.8%). Similar data were observed in a population of 500 homeless patients in Portugal (13) and in Germany (14) both at 24%. While in Stockholm, a prevalence of 12% was reported in 1704 homeless patients receiving hospital care (15). In Japan it was 3.5% (≈114 homeless) (16). Conversely, a prevalence of 50% was observed in data from London with 560 homeless men (17) and 57% based on the Edinburgh survey (≈44) (18). The prevalence reached a record high of 80% in a French study using epidemiological measures (≈1200 homeless men) (19) and 88% (with a mean of 3.5 diagnoses per participant) in the United States of America (USA) (≈99) (20). These examples illustrate the extensive range and diversity of conditions analyzed, emphasizing the clinical relevance of data on the presence of the PD’s among homeless populations.

A substantial number of psychiatric disorders are well documented in homeless populations (13). Homeless people with multiple diagnoses have greater mental health needs and worse general health determinants (9, 13). A general synthesis of the prevalence of comorbid psychiatric disorders in homeless patients, according to reports reveals the following figures: psychotic disorders among 4.4%–57% (8, 13, 14, 16); major depression among 11.4% (8); bipolar disorders among 11.4%–17.5% (8, 16); alcohol dependence among 14.3%–37.9% (8, 13, 14, 16); drug dependence among 14.3%–34% (8, 13, 14, 16); acute stress reaction among 23%–24% (13, 14); and anxiety disorder among 2.3% (16). These references are based on studies carried out in the USA, France, Japan, Portugal, and Germany.

The findings from ten-year records of homeless patients attending emergency services (≈2750) in the USA show greatly increased rates of admissions for alcohol, substance abuse and psychiatric-related problems, particularly for schizophrenia (Odd Ratio, OR:16.6) and PD (OR:15.4) (21). Lipton’s study (22) of homeless patients at a hospital emergency department supported this finding: 96.6% of this patient population had a previous psychiatric hospitalization, 72% had been diagnosed with schizophrenia, and the second most common diagnosis was PD (13.3%). In another study conducted in Portugal by Bento and Barreto (23) with a reference population of 511 homeless patients, 94% of the overall sample included patients with psychiatric disorders, excessive alcohol/drug consumption, and PD’s.

A small number of studies have recognized the existence of specific PD among the homeless, including antisocial, schizoid, dependent, and borderline PD’s (12). Connoly et al. (12) reported that the rates of specific Axis II disorders exceeded the rates of specific Axis I disorders by 50%. However, few studies have conducted systematic assessments of the full range of PD or evaluated their relationship with the Diagnostic and Statistical Manual of Mental Disorders, fourth edition (DSM-IV) Axis I diagnoses, often relying on unstructured assessments (11, 12). In this brief review, we identified only seven studies addressing the full range of PD diagnoses (7, 12, 19, 20, 24–26). For data systematization *vide* **Table 1**, where we also included the geographic area and its respective climate type (27). All studies were done in both sides of North Atlantic Ocean: four in the East coast of the United States of America and three in the Western part of the European Union. We believe the harder winters in humid continental climates at the states of Massachusetts, New York and Connecticut may have an influence in how homeless people live, somehow different from what happens in Spain with Hot Summer Mediterranean and France Oceanic climate types (27). On the other hand, the cultural differences may not have such an impact, as all studies were performed in the prevalent and quite accepted homogeny of the Western world. Culturally speaking the White Anglo-Saxon Protestant (WASP) culture in the northeastern USA has little contrast with the Latin catholic culture in the western EU, in the impact how psychiatric homeless people live in the streets. Furthermore, in a systematic review (10) which is based on analysis of five of these studies (*i.e*., 7,12,24,25,26), it is globally reported that the most prevalent PD diagnoses in homeless populations were paranoid (14%–74%), avoidant (14%–63%), borderline (6%–62%), and antisocial (4%–57%).

**Table 1:** General data of the specific personality disorders among the homeless people in the studies included in the present review.

Publication	*N*	*Mean* Age	Sex	Personality disorders(%)	Psychiatry disorders(%)	Area (Climate)
Bassuk et al. (25)	80	27	100% female	Axis II total(71) Schizoid(3) Antisocial(4) Borderline(6) Histrionic(1) Narcissistic(4) Dependent(24) Other(13) Atypical(10) Mixed(4) Passive-aggressive(4)	Axis I total(27) Affective disorders(10) Substance abuse(9) Mental retardation(5) Schizophrenia(3)	Massachusetts, United States of America (Humid Continental)
Ball et al. (24)	52	38	94% male	Paranoid(74) Schizoid(42) Schizotypal(56) Antisocial(47) Borderline(51) Histrionic(23) Narcissistic(35) Avoidant(63) Dependent(12) Obsessive-compulsive(61) Cluster A(88) Cluster B(74) Cluster C(85)	Illicit substance abuse(50) Alcohol abuse(50)	New York, United States of America (Humid Continental)
Connolly et al. (12)	60	41	68% male	Paranoid(73) Schizoid(65) Schizotypal(43) Antisocial(57) Borderline(62) Histrionic(20) Narcissistic(57) Avoidant(50) Dependent(25) Obsessive-compulsive(57) Cluster A(92) Cluster B(83) Cluster C(68)	Substance dependence(62) Anxiety disorders(62) Mood disorders(55) Psychotic disorders(20)	New York, United States of America (Humid Continental)
Combaluzier et al. (19)	212	27	100% male	Axis II total(95) Paranoid(1) Schizoid(9) Schizotypal(1) Antisocial(42) Borderline(16) Histrionic(4) Avoidant(4) Dependent(12) Obsessive-compulsive(2) Other(5)	Drug dependence(100)	France, European Union (Oceanic)
Samuel et al. (20)	99	41	57% male	Axis II total(86) Paranoid(55) Schizoid(39) Antisocial(≈20) Borderline(≈20) Histrionic(9) Narcissistic(41) Dependent(5) Obsessive-compulsive(58)	Axis I total(85) Mood disorders(67) Substance use(66) Anxiety disorders(59) Post-traumatic stress(25) Psychotic disorders(19)	Connecticut, United States of America (Humid Continental)
Salavera et al. (7)	89	39	100% male	Paranoid(14) Schizoid(19) Schizotypal(16) Antisocial(26) Borderline(9) Histrionic(79) Narcissistic(9) Avoidant(14) Dependent(20) Obsessive-compulsive(23) Aggressive(15) Passive(9) Self-defeating(9)	–	Spain, European Union (Hot Summer Mediterranean)
Salavera et al. (26)	196	<40	–	Axis II total(79) Schizoid(21) Antisocial(26) Dependent(22) Obsessive-compulsive(26)	Attention deficit hyperactivity disorder	Spain, European Union (Hot Summer Mediterranean)

Other studies (28, 29) identified the most prevalent diagnoses among the homeless population as substance abuse and PD’s, including antisocial PD (28). This population had higher rates of alcohol abuse disorder (men), drug abuse disorder (women), and antisocial PD (both men and women) (28). The only diagnosis that was more prevalent in homeless clinics than in communities was antisocial PD (28). Similarly, Caton (30) reported a significantly higher number of homeless individuals with a concurrent diagnosis of antisocial PD and borderline PD (9).

However, some authors have argued that among the homeless, many of the features of antisocial personality may be artifacts of homelessness and that strict application of the diagnostic criteria may be insensitive to nurture factors (11). A study among 600 homeless individuals (31), found that data support the appropriateness of the diagnosis of antisocial PD among these populations. Most adult symptoms of antisocial PD were associated with the number of childhood conduct disorder symptoms (nature), and the onset of symptoms usually preceded the onset of homelessness (31).

Other important findings suggest a higher-than-normal prevalence of schizoid PD potentially playing a role in treatment engagement and chronicity of homelessness (32) and schizotypal PD (33). Finally, the diagnosis of emotionally unstable PD appeared to be associated with homelessness referrals to an acute young adult psychiatric unit (34). Still, a single case report of a schizoaffective homeless man with a previous diagnosis of *haltlöse* PD highlights the need for more studies examining PD Not Otherwise Specified (NOS) (35).

### 3.2 Personality disorders related to risk factors among homeless people and associated psychological and behavioral outcomes

Personality disorders (OR: 2.2) are identified as a risk factor associated with an increased risk of homelessness. They along with severe psychiatric disorders, substance abuse, and pathological gambling constitute the most significant modifiable factor, as determined by a USA big data study examining risk and protective factors for homelessness (36).

Findings of a French research (19) (≈1200 homeless men) lead to the conclusion that PD increases the risk of substance abuse, subsequently increasing the risk of homelessness. This dual diagnosis has a high impact on homelessness. The comorbidity of drug abuse and PD multiplies the risk of homelessness by a factor of 7, accounting for 46% of the cases. Conversely, the association between PD and homelessness multiplies the risk of drug abuse by a factor of 13, accounting for 3/4 of drug abuse cases (19). Moreover, PD’s appear to have a basic role in the etiopathology of such a morbid constellation because the frequency of their observation is independent of the association between homelessness and drug abuse (19). Another study (37) highlighted the association between homeless individuals and a specific group of people - those with serious substance misuse and PD (39.3%).

In a two-year longitudinal study conducted in Canada (38) young adults experiencing first-episode psychosis, within the homeless group were more likely to have childhood abuse, forensic history, non-affective psychosis, negative symptoms, substance use disorder, and the DSM-IV Cluster B PD (referred to as bad PD). It is also associated with poorer symptomatic and functional outcomes despite having more long-acting injectable antipsychotics, community treatment orders, and hospitalizations (38). Poor prognostic factors were related to Cluster B PD in intensive outreach services dedicated to homeless youth experiencing first-episode psychosis and addiction in another longitudinal study (39).

Studies have reported that high rates of deliberate self-harm and suicide in the homeless are related to high rates of psychiatric disorders found in this population, predominantly schizophrenia (40). Among homeless individuals, those exhibiting high rates of drug and alcohol abuse and PD were most often those without a stable residence. They were more likely to be male, single, unemployed, recent victims of violence, prone to have violent behavior toward others (40), a criminal record, and to have a PD (40, 41), as well as increased mortality from all causes (40).

Data focusing on gender and prevalence of psychiatric disorders among hospitalized homeless patients (15) revealed the following. Homeless women were at a higher risk for psychiatric disorders than homeless men (1.20), and younger homeless women had the highest risk (2.17). Alcohol use disorders were equally common, but women had a higher prevalence of drug use disorders (1.32). Women were at higher risk of schizophrenia (2.79) and PD’s (2.73). Indices of low quality of life include middle-aged homeless women living in temporary housing with criminal records, PD, and substance use disorders (42). Risk factor evaluation for homelessness among patients with severe psychiatric conditions (43, 44) show distinct patterns. Among homeless women with schizophrenia, higher rates of concurrent diagnosis of alcohol abuse, drug abuse, and antisocial PD, including less adequate family support (43); Among homeless men with schizophrenia, there was widespread concurrent substance abuse and antisocial PD (42%), and 72% had a history of incarceration (44). In addition to childhood antecedents, data indicate that drug abuse and antisocial PD preceded homelessness (44). Notably, 4/5 male patients experiencing homelessness had a triple diagnosis – concurrent schizophrenia, substance abuse, and antisocial disorder–indicating the presence of these traits even before adolescence (45). Consequently, inadequacies in psychiatric service discharge planning are most apparent among homeless men with heavy tri-morbidity (30).

Furthermore, antisocial PD is associated with illegal economic activities (selling drugs, theft, and prostitution) for income generation among the homeless (46). This association extends to youth homelessness (47), which in combination with arrest history serves as a risk factor for recurrent homelessness (48). Moreover, it is coupled with gambling disorder (49), violent behavior (50), and HIV risk in homeless individuals (51).

Engaging in survival sex is over-represented within homeless populations (52), and data show robust associations with symptoms of borderline PD, childhood abuse, and post-traumatic stress disorder among homeless women (52, 53), suggesting that older individuals with high levels of impulsivity symptoms may be especially at risk (52). Similar approaches among homeless men have shown that risky sexual behavior is accompanied by common symptoms of PD’s and predicts treatment outcomes and suboptimal achievements in health-promoting or prosocial behaviors (54–56). Nevertheless, a risk index comprising key symptoms of antisocial/borderline disorders plays an essential role in sexual risky behaviors in both sexes (57).

Another focus comes from the sheltered homeless families, with suspicions of probable child abuse or neglect, where it is observed that 1/4 of the mothers had the presence of major clinical psychiatric syndromes and 70% of the mothers had PD (25). In this follow-up on the relationship between homelessness, mental health, and motherhood, the findings showed that 2/3 of the young mothers with children in their care met the criteria for lifetime antisocial PD (58) and borderline PD (59). These were further associated with criteria for lifetime major depressive episodes, post-traumatic stress disorder, and drug abuse (58).

### 3.3 Personality disorders in relation to clinical and intervention outcomes among homeless people

The risk factors for unplanned hospital admission in homeless individuals have been reported (60). Enduring psychiatric conditions and/or PD (OR:3.84), establish themselves as the greatest risk factors increasing the likelihood of admission by almost four-fold. This impact on the likelihood of poor physical health outcomes, potentially because of a lack of engagement or late presentation to services. When homeless patients access health services, maladaptive behaviors are often associated with poor attendance, reduced effectiveness of therapeutic alliances, failure to follow through on referrals, noncompliance with medications for medical or psychiatric symptoms, and suicidal behaviors (24).

A congregation is characterized by high rates of PD’s among profiles of homeless individuals, and high overall medical service use (29, 42). In contrast, homeless patients who underuse mental health services (24, 30, 61) are more likely to receive psychiatric treatment in hospitals rather than in outpatient services and have inadequately planned psychiatric hospital discharge. This is more likely if they have comorbidities of schizophrenia, substance abuse, and antisocial PD (30).

PD’s significantly influence the failure of homeless people to adhere to treatment (12). When homelessness and PD coexist, the likelihood of treatment non-adherence increases. Notably, Cluster B PD are associated with avoiding permanence in the treatment process, while Cluster C PD (referred to as sad PD) are connected to favored treatment adherence and improved prognosis (7). Specifically, borderline and passive-aggressive PD’s (another type of PD NOS) were reasons for treatment abandonment in 100% of the patients. Additionally, patients with antisocial, obsessive-compulsive, or paranoid PD seemed to be related to treatment abandonment (7).

Concerning factors associated with health service use, the literature reports that, in young homeless people, the presence of PD (OR:4.9) was estimated to be one of the factors that improved lifetime health service utilization or follow-up (62).

In addition, the presence of PD in the homeless is linked to several factors: poorer rates of adherence and completion of psychiatric and therapeutic treatment (63), worse outcomes for treatment of depression, and an increased risk of deliberate self-harm (8), insecure types of attachment that may impact intervention strategies (10), acting as a barrier to the formation of a therapeutic alliance (64), influencing the benefits of therapeutic approaches (19), and contributing to comorbidity in dual diagnosis that may benefit from pharmacist intervention to address medication-related problems (65).

### 3.4 Challenges linked to personality disorders in the assessment, treatment, and intervention for homeless people

Traditional models of service delivery in Western countries, which focus on those with severe psychiatric disorders, may not meet the mental health needs of most homeless individuals with substance dependence and PD (8).

Authors such as Bassuk et al. (25) drew attention to PD as a diagnosis of social dysfunction and did not consider the influence of environmental factors extrinsic to the organization of personality, such as poverty, racism, and gender bias. The criteria for these disorders are descriptions of behavioral disturbances that are long-term and predate homelessness. Thus, the labels should primarily be used to indicate severe functional impairment and the need for help rather than implying strict causality (25).

According to Ball et al. (24), some of the paranoid, hostile, and bizarre symptoms of the homeless may be adaptive or at least understandable, given the extreme challenges of living on the streets or in a shelter. Although a diagnosis of PD requires evidence of the early onset of maladaptive traits, it is difficult to rule out the possibility that some Cluster A PD (referred to as mad PD) may be better understood as a consequence rather than as a cause of homelessness (24).

Furthermore, there are very few studies on homeless populations that have systematically assessed the full range of PD’s using appropriate and rigorous methodologies and evaluation criteria for PD assessment, thus concluding that this is an important gap and challenge (11, 12).

Mental health services for the homeless facing particularly high levels of factors associated with suicide and homicide, a significantly higher prevalence of PD, and targeting poor compliance and complexity of disorders, require significant input from multidisciplinary mental health team members (66). Managing mood in this population remains a major challenge and nonpharmacological treatments (including complementary agents and psychosocial interventions) should be evaluated to address this issue (50). Additionally, data indicate that PD’s in the homeless are probably more common among women emphasize an important factor for social and healthcare services to bear in mind (15).

Although it remains unclear whether this group of patients is amenable to individual or group psychotherapy, they have a profound need for other social services, and some may benefit from counselling or pharmacotherapy to help improve adaptive functioning or reduce Axis I symptoms (24).

Clinicians treating homeless outpatients may benefit from having special facilities for the diagnosis and management of PD’s and substance abuse along with expertise in other comorbid psychiatric disorders (28). Homeless treatment seekers might benefit from the specialized programming and services of clinicians who are especially proficient in recognizing and treating the disorders best represented in these populations, which are notoriously difficult to manage (28).

Highlighting that, early trauma experiences have lifelong consequences, so complex trauma, appears to be intrinsic related to psychopathology and personality disorders in the homeless persons (8, 10, 29). Within the developing of Trauma-Informed Care (TIC), an awareness of these issues in a range of services, should improve the establishment of Psychologically Informed Environments (PIE) first taking place in re-designed facilities for homeless people (29, 67).

Nevertheless, we cannot be sure if paranoid, avoidant, or even obsessive-compulsive personality disorders are cause or consequence of being homeless. All these three personality disorders may be mimicked by survival street behavior. For example, a person experiencing homelessness can perfectly assume an avoidant and/or paranoid to avoid conflicts with other people. On the other hand, obsessive compulsive personality disorder can be mimicked by a hoarding behavior, especially with food or other essential items, in order to increase the chances of survival (10). Regarding the anti-social PD is even more difficult to distinguish cause/nature from consequence/nurture: was the homeless born genetically vulnerable to psychopathy or it was the street hard life than made that person a sociopath?

Further investigations are needed regarding homeless people with psychiatric disorders and their treatment, particularly those with multiple diagnoses that have worse health determinants (13).

As a main conclusion and guideline for further research, Salavera et al. (7) viewed PD as a prognostic factor in treatment. Therefore, reintegration processes, and prevention strategies must be clearly established, considering the subject’s personality as a basic element, and providing an individualized therapeutic process (7, 29). Knowledge of these personality traits should be used to advocate for better healthcare services for supporting homeless individuals (7).

## 4 Conclusion

The homeless population suffers from major diagnostic variability and the diagnosis of PD’s is still evolving, contributing to difficulties in diagnosis, assessment, and treatment. However, further studies are warranted and should focus more on the causes and effects of events. It is important to highlight as a limitation that the percentages of personality disorders analyzed in this review are based on studies with a disparate number of participants.

Does PD predispose individuals to homelessness? Does precocious homelessness contribute to PD? Does antisocial psychopathy increase the probabilities of homelessness? Or is it the homeless lifestyle that produces antisocial sociopaths? Do obsessive-compulsive personality hoarding habits lead to people being expelled from home and condemned to street life? Or is it homelessness that produces hoarding behavior for better chances of surviving on the streets? How many PD’s are NOS, such as *haltlöse* or passive-aggressive, and are underestimated among homeless people? What is the importance of attachment dysfunctions? What is the role of PD’s secondary to organic conditions such as seizures or epilepsy, which is also common among homeless people (68)?

A challenge for the future is to raise clinical awareness and optimize research knowledge, assessment, and interventions for PD’s among homeless individuals with comorbid psychiatric disorders and drug abuse. These individuals are often referred to as super-difficult patients, the subjects of Marontology, a new, unborn, medical specialty, suggested after the Greek word *maron-tos*, which means unwanted (69).

## Author contributions

JH-C: Funding acquisition, Writing – original draft, Writing – review & editing. JGM: Writing – original draft, Writing – review & editing.
